# Differences in the Experience of Caregiving Between Spouse and Adult Child Caregivers in Dementia With Lewy Bodies

**DOI:** 10.1093/geroni/igz027

**Published:** 2019-09-04

**Authors:** Taylor Rigby, Robert T Ashwill, David K Johnson, James E Galvin

**Affiliations:** 1 Department of Psychology, University of Kansas, Lawrence; 2 Georgia Institute of Technology, Department of Psychology, Atlanta; 3 Department of Neurology, University of California at Davis Health Sciences, Walnut Creek; 4 Comprehensive Center for Brain Health, Charles E. Schmidt College of Medicine, Florida Atlantic University, Boca Raton

**Keywords:** Burden, Caregiving, Depression, Family caregivers, Grief, Informal, Quality of life, Social networks, Social Support, Well-being

## Abstract

**Background and Objectives:**

Dementia caregiving has been associated with increased burden, depression, grief, a decreased sense of well-being and quality of life, and a weakening of social support. Little is known about the experience of caregiving in Dementia with Lewy Bodies (DLB). The present study examines differences in the caregiving experience of spouse versus adult child caregivers of individuals with DLB.

**Research Design and Methods:**

In this cross-sectional analytic study of spouses (*n* = 255) and adult children (*n* = 160) caregivers of individuals with DLB, participants completed an online survey of burden, grief, depression, well-being, quality of life, and social support.

**Results:**

Adult child caregivers were more likely to care for women (*p* < .001) and see the care recipient less often (*p* < .001) than spouses. Adult child caregivers reported lower quality of life (*p* < .001) and more caregiver burden (*p* < .009), but also greater social support (*p* < .001) than spouses. Between group analyses of caregiver type by disease severity demonstrated that spousal caregivers experience greater grief with advancing disease (*p* = .005), while adult child caregivers increase social support with advancing disease (*p* < .001).

**Discussion and Implications:**

Spouses and adult children experience DLB caregiving differently. This was explained by the younger age of the adult child caregiver, frequency of contact with the care recipient, and differences in the care recipient’s characteristics, frequency of neuropsychiatric symptoms, and disease severity. DLB caregiver support for this population should target psychoeducation for complicated neuropsychiatric symptoms in the care recipient. Screening all DLB caregivers for burden, grief, and depression is suggested to identify those that may benefit most from intervention. Spouses specifically may benefit from interventions that target increasing social support, while adult child caregivers may benefit from interventions aimed at mitigating burden and improving quality of life.

**Translational Significance**

Adult children and spouses experience caregiving differently for Dementia with Lewy Bodies (DLB). Clinicians should be aware that the experience of increased burden and grief for DLB caregivers may reflect differences in social support and the care recipient’s characteristics, frequency of neuropsychiatric symptoms, and disease severity. Effective interventions for DLB caregivers should take into account the differential perceptions and needs of spouses and adult child caregivers.

## Background and Objectives

Caregiving for people with dementia has been associated with increased burden, depression, grief, a decreased sense of well-being and quality of life, and a weakening of social support (Anderson et al., 2013; Etters, Goodall, & Harrison, 2008; Pinquart & Sörensen, 2011). Dementia with Lewy bodies (DLB) is estimated to affect 1.4 million people in the United States (Lewy Body Dementia Association, 2019). DLB has many shared features with other forms of dementia such as Alzheimer’s disease (AD); however, the onset and disease course of DLB is distinct (Ferman et al., 2013; Karantzoulis & Galvin, 2013; McKeith et al., 2004; Morra & Donovick, 2014; Ricci et al., 2009; Tarawneh & Galvin, 2007). DLB is marked by the early onset of recurrent visual hallucinations, parkinsonism, rapid eye movement sleep behavior disorder, and spontaneous alterations in concentration and attention known as fluctuating cognition; this constellation of symptoms is unique to DLB and are central to establishing a diagnosis of DLB (Ferman et al., 2013; Karantzoulis & Galvin, 2013; McKeith et al., 2004; Morra & Donovick, 2014; Ricci et al., 2009; Tarawneh & Galvin, 2007). When compared with AD, those with DLB have a shorter survival time from disease onset and in terms of overall mortality (Williams, Xiong, Morris, & Galvin, 2006). DLB is also associated with a shorter time to institutional placement than AD (Tarawneh & Galvin, 2007). Despite differences between DLB and AD in presentation and disease course, extant studies have focused primarily on caregivers of patients with AD and these findings have been extrapolated to cover caregivers for patients with other forms of dementia. There are relatively few studies that specifically examine the experience of caregivers of DLB.

### Caregiver Burden

Burden is a complex process that is context driven and affected not only by the primary stressors of the care recipient’s disease (e.g., decline in cognition, physical functioning, behavioral problems) but also the resulting secondary stressors—whereby stress permeates other areas of a caregivers’ life outside of their caregiving role (Pearlin, Mullan, Semple, & Skaff, 1990). Caregiver burden incorporates the overall experience dealing with various physical, psychological, emotional, social, and financial stressors (Kasuya, Polgar‐Bailey, & Takeuchi, 2000) and reflects the level of dependency of the person with dementia on their caregiver (Brodaty & Donkin, 2009); in DLB this includes the frequency and severity of noncognitive symptoms (neuropsychiatric features, extrapyramidal features, autonomic and sleep features, etc.). Studies examining how the caregiver’s relationship with the care recipient (e.g., spouse vs adult child) impacts caregiver burden have yielded mixed results in the literature. Some studies have shown that spouse caregivers of dementia report more burden than adult child caregivers (Andrén & Elmståhl, 2008; Galvin et al., 2010; Hong & Kim, 2008; Rinaldi et al. 2005), while other studies show the reverse (Coen, O’Boyle, Coakley, & Lawlor, 2002; Conde-Sala, Garre-Olmo, Turró-Garriga, Vilalta-Franch, & López-Pousa, 2010a; Molyneux, McCarthy, McEniff, Cryan, & Conroy, 2008). In one study examining the burden of caregiving for those with DLB and Parkinson’s disease with dementia, spousal caregivers had higher burden scores than nonspousal caregivers (Galvin et al., 2010).

### Caregiver Grief

Grief results from perceived loss, and can manifest in psychological, physical, social, behavioral, and affective forms (Marwit & Meuser, 2002; Ott, Sanders, & Kelber, 2007). Caregiver grief and bereavement are associated with an increased risk of mortality, and decrements in physical and mental health (Etters et al., 2008; Stroebe, Schut, & Stroebe, 2007). There is limited study of caregiver grief in DLB. A study of grief reactions in caregivers of individuals with AD found no difference between spouses and adult children when the care recipient lived in the family home, but spouses reported more grief than adult children once the care recipient was placed in a facility outside the home (Ott et al., 2007). Marwit and Meuser (2002) found that spouses reported more overall grief than adult child caregivers of AD, and that grief responses differed over the care recipient’s disease course; adult child caregiver grief reached a peak in the moderate stages of patient decline, while spousal grief increased linearly and was greater overall to that of adult children.

### Caregiver Depression

Depression has not been well studied in caregivers of DLB. A meta-analysis found that caregivers report more depression than noncaregivers (Pinquart & Sörensen, 2011). DLB and AD caregivers have been shown to report a similar frequency of depression (Lowery et al., 2000). The extant literature suggests that spouse caregivers report more depression symptoms than adult child caregivers when caring for a family member with dementia, but the results are mixed (Covinsky et al., 2003; Lou et al., 2015; Pinquart & Sörensen, 2011; Schulz & Sherwood, 2008 vs Watson et al., 2011).

### Psychological Well-being

Numerous studies have shown that caregiving has a negative impact on the psychological well-being of caregivers (Etters et al., 2008; Pinquart & Sörensen, 2011). Psychological well-being captures a number of attributes including positive self-regard, mastery, quality relations with others, growth and development, sense of purpose, and autonomy associated with current life situations (Ryff & Keyes, 1995). This construct has not been previously explored in DLB.

While contrasting results have been reported in the literature, a meta-analysis finds that spouse caregivers, across studies, report lower levels of psychological well-being than do adult children (Pinquart & Sörensen, 2011).

### Quality of Life

The presence of cognitive and noncognitive symptoms (neuropsychiatric features, extrapyramidal features, autonomic and sleep features, etc.) and functional deficits in the care recipient have a negative impact on the quality of life of dementia caregivers (Conde-Sala, Garre-Olmo, Turró-Garriga, Vilalta-Franch, & López-Pousa, 2010b). Quality of life integrates physical health, psychological state, perception of autonomy, social relationships, personal beliefs, and one’s relationship with environmental events (Hawthorne, Richardson, & Osborne, 1999; Rabins & Kasper, 1997) and can be placed in context of the respondents culture and value system, goals, expectations, and standards (WHO, 1997). Caregivers of DLB have been found to report a lower quality of life than caregivers of AD (Thomas et al., 2006). Age also appears to impact the perception of quality of life. It has been found that older people, when compared with younger people, reported fewer negative emotional experiences and greater emotional control (Gross et al., 1997). Similarly, in the dementia caregiver literature, older caregivers report being more satisfied with their life and having a lower prevalence of frequent mental distress than younger caregivers despite having greater age-related physical health concerns (Anderson et al., 2013), and spouses perceive the care recipient’s quality of life more positively than do adult child caregivers (Conde-Sala et al., 2010b).

### Social Support and Social Networks

Having the perception of good social support has been shown to be related to greater life satisfaction (Haley, La Monde, Han, Burton, & Schonwetter, 2003; Hämmerling, Ludwig, & Wendel, 2008; Tomomitsu, Perracini, & Neri, 2014) and fewer depressive symptoms in caregivers (Clay, Roth, Wadley, & Haley, 2008). Social support has been defined as the perception of being cared for, loved, esteemed, and a member of a network of mutual obligations (Cobb, 1976). Adult children caregivers of dementia show a greater tendency to make use of formal community resources and services (e.g., family physicians, nurses, and social workers; Robinson, Buckwalter, & Reed, 2005) and informal social support (e.g., family members, friends, and neighbors) than spouses (Pinquart & Sörensen, 2011).

### Current Study

There are few studies that examine the DLB caregiver experience or take into account multiple constructs associated with that experience. There are even fewer that examine the potential differences between being a spouse versus adult child caregiver of DLB. The lack of literature may be contributing to poorer outcomes for caregivers of DLB (Galvin et al., 2010). The purpose of the present study was to examine differences in the caregiving experience for spouses versus adult child caregivers when caring for individuals with DLB. The presence of neuropsychiatric symptoms (such as recurrent hallucinations, delusions, sleep disorders, and depression) in addition to cognitive decline and parkinsonism likely adds to caregiver burden, grief, and depression (Ferman et al., 2013; Galvin et al., 2010; Holley & Mast, 2010; Leggett et al., 2011; Ricci et al., 2009; Schulz & Sherwood, 2008). Based on research conducted with other caregivers of dementia, there is more evidence to support that spouse caregivers report more depression and a lower rated sense of well-being than adult child caregivers; however, the dementia caregiving literature is mixed as regards the impact of burden and grief on spouse caregivers versus adult children. Due to the lack of literature exploring differences among caregivers of DLB and the mixed results in the larger dementia caregiving literature, these analyses should be considered largely exploratory; thus, our hypothesis was that spouses and adult children would experience caregiving for DLB differently, and we would see differential responses patterns for burden, grief, depression, and well-being, by caregiver type and stage of disease. Given that these were exploratory analyses, the nature of these differences was not specified. Despite having more physical health concerns, older adults rate quality of life higher for themselves and the care recipient when compared with younger caregivers (Anderson et al., 2013; Conde-Sala et al., 2010b). Thus, it was hypothesized that spouses would report greater quality of life than adult children. Because adult children caregivers of dementia show a greater tendency to make use of community resources (Robinson et al., 2005) and report more informal social support than spouses across studies (Pinquart & Sörensen, 2011), it was hypothesized that adult children would report having more social support and larger social networks.

## Research Design and Methods

### Participants

Potential participants were contacted with the assistance of the Lewy Body Dementia Association and several partner organizations. Recruitment information was sent out to individuals on caregiver e-mailing lists of these organizations and was listed on their webpages and social media sites. The e-mail provided potential participants with information about the study and instructions on how to participate. Interested individuals were invited to participate in the study by following a link to the online survey. The participants who were selected for this study were either a spouse or an adult child caregiver of living individuals with a diagnosis of DLB.

### Procedures

A 230-question survey was created and made available online for 3 months using Survey Monkey (www.surveymonkey.com, Palo Alto, CA). Eligible participants accessed the survey via an e-mailed link with no time limit to complete (participants could save and return to the survey later). All collected personal health information remained confidential and all data was deidentified during analyses. This study was approved by Internal Review Board at the New York University Langone Medical Center.

### Measures

#### Sociodemographic

Participants were asked to provide information about their social and demographic characteristics and that of their care recipient with DLB. This information is presented in Table 1.

**Table 1.** Sample Characteristics

	Spouse	Adult child	*p* value
Caregiver characteristics	(*n* = 255)	(*n* = 160)	
Age, years	65.9 (8.7)	52.6 (7.7)	<.001**
Sex, % Female	87.4	91.1	.151
Education, % >12 years	88.6	90.7	.932
Race, % White	98.0	95.6	.557
Ethnicity, % Hispanic	0.4	3.8	.022*
Marital Status, % Married	99.6	68.8	<.001**
Lives with patient, %	88.9	36.3	<.001**
Sees patient daily, %	85.3	44.5	<.001**
Primary caregiver, %	97.6	73.0	<.001**
Care recipient characteristics			
Age, years	71.2 (8.0)	81.8 (7.2)	<.001**
Sex, % Male	88.6	34.6	<.001**
Education, % >12 years	78.0	44.5	<.001**
Race, % White	96.1	95.6	.872
Ethnicity, % Hispanic	0.8	3.1	.008*
Marital Status, % Married	99.6	33.3	<.001**
Housing situation, %			<.001**
Private residence	85.9	62.5	
Retirement community	2.7	3.8	
Assisted living facility	3.1	13.1	
Skilled nursing facility	8.3	20.7	
Duration of disease, years	3.8 (2.4)	3.6 (2.7)	.441
Stage of disease, %			<.001**
Mild	8.6	4.4	
Moderate	63.9	46.9	
Severe	27.5	48.8	
QDRS	14.8 (6.3)	18.5 (6.7)	<.001**
CDR-SB	9.7 (4.1)	11.7 (4.2)	<.001**
Neuropsychiatric Symptom Frequency	37.2 (13.9)	41.6 (17.2)	.006*
Care recipient Quality of life^a^	26.8 (5.9)	24.9 (5.3)	.001**

*Note*: Analysis of variance was used to examine group differences on continuous variables; Chi square statistics were used to examine group differences on categorical variables; when applicable, mean, standard deviation, % of sample, and *p*-values reported; disease severity (mild, moderate, severe) characterized by caregiver global ratings of disease severity. CDR-SB = Clinical Dementia Rating Sum of Boxes; QDRS = Quick Dementia Rating System.

^a^Quality of life of the care recipient as rated by the caregiver.

*Trend to significance *p* < .05.

**Significant after correction for multiple comparisons *p* ≤ .003.

#### Care recipient’s disease stage

The Quick Dementia Rating System (QDRS) was used to determine the presence of impairment and, if present, rate its severity (Galvin, 2015). The QDRS has a high correlation with the Clinical Dementia Rating (CDR; Morris, 1993), and has high reliability (α: 0.86–0.9; Galvin, 2015). The QDRS rates cognitive function in 10 domains: memory and recall, orientation, decision-making and problem-solving abilities, activities outside the home, function at home and hobbies, toileting and personal hygiene, behavior and personality changes, language and communication abilities, mood, and attention and concentration. Participants answer questions pertaining to the care recipient’s level of functioning (e.g., “Solves everyday problems; handles business and financial affairs well, judgment and decision making consistent with past performance”; “Mild but definite impairment of function at home; more difficult chores or tasks abandoned; more complicated hobbies and interests given up”; “Severe comprehension deficits; no intelligible speech”) with scores ranging from 0 (indicating no impairment) to 3 (indicating greater impairment), with higher total scores indicating more significant cognitive and functional impairment (i.e., greater disease stage and severity). The QDRS can be used as a sum of scores in the 10 domains (range 0–30), or the first six domains can be used to derive a CDR sum of boxes (CDR-SB) and a global CDR (using the published CDR scoring rules; Morris, 1993). Both the QRDS score and a CDR-SB were used in the analyses. In addition, the respondents were asked to provide a global rating of the stage of the patient as mild, moderate, or severe taking into account all aspects of disease (e.g., cognition, function, behavior, mood). This strategy has been successfully used to provide global disease ratings in survey studies in DLB (Galvin et al., 2010) and Frontotemporal degeneration (Galvin, Howard, Denny, Dickinson, & Tatton, 2017) and corresponds well with other outcome measures.

#### Frequency of care recipient’s neuropsychiatric symptoms

The dementia care recipient’s overall frequency of neuropsychiatric symptoms was assessed using the Revised Memory and Behavioral Problems Checklist (RMBPC; Teri et al., 1992). The RMBPC contains 24 statements and has high reliability (α = 0.84; Teri et al., 1992). Participants answer questions pertaining to the frequency of caregiver observed memory and neuropsychiatric symptoms in the care recipient (e.g., “Trouble remembering recent events”; “Being aggressive to others verbally”; “Appearing sad or depressed”). Questions are answered using a five-point Likert scale ranging from *never occurred* to *occurred daily or more often*, or *do not know/not applicable*. Higher scores on the RMBPC indicate greater levels of caregiver perceived neuropsychiatric symptoms in the care recipient.

#### Caregiver burden

Caregiver burden was measured using a 12-item abridged version of the Zarit Burden Interview (ZBI). The ZBI is one of the most commonly used measures of caregiver burden; it was originally developed for use with caregivers of AD but has been validated for use in DLB and has a high combined reliability (α = 0.86; Leggett et al., 2011). The ZBI is comprised of questions asking caregivers about their experiences of emotional, physical, and social strains or difficulties that result from their role as a caregiver (e.g., “Do you feel that because of the time you spend with the patient that you do not have enough time for yourself”; “Do you feel strained when you are around the patient”; “Do you feel you could do a better job in caring for your relative”). In the ZBI, participants respond to questions using a five-point Likert scale ranging from *never* to *nearly always*. Higher scores indicate higher perceived levels of burden.

#### Caregiver grief

Caregiver grief was measured using the Marwit-Meuser Caregiver Grief Inventory Short Form (MM-CGI-SF). The MM-CGI-SF was designed to measure grief in caregivers of persons with progressive dementia (Marwit & Meuser, 2005). The MM-CGI-SF is composed of 18 statements and assesses caregiver grief on three factors that all have high reliability (Factor 1, α = 0.83; Factor 2, α = 0.80; Factor 3, α = 0.80; Marwit & Meuser, 2005). Questions relate to grief experienced as a result of dementia caregiving (e.g., “My friends simply don’t understand what I’m going through”; “I long for what was, what we had and shared in the past”; “I feel I am losing my freedom”). Participants respond using a five-point Likert scale ranging from *strongly disagree* to *strongly agree*. Higher scores indicate greater feelings of grief. As no differences were found for the three factors, only the global Caregiver Grief score is reported.

#### Caregiver depression

Caregiver depression was measured using the Patient Health Questionnaire 2-item Depression Scale (PHQ2). The PHQ-2 comprises the first two questions of the PHQ-9 that was designed as a screening tool for unipolar depression (Kroenke, Spitzer, & Williams, 2003). The PHQ-2 has good agreement with independent mental health professionals and moderate reliability (α = 0.65; Spitzer, Kroenke, Williams, & Patient Health Questionnaire Primary Care Study Group, 1999). Participants are asked to rate, on two questions, how often they have experienced symptoms of depressed mood and anhedonia over the past 2 weeks: (1) “Little interest or pleasure in doing things;” 2) “Feeling down, depressed, or hopeless”. Participants respond using a four-point Likert scale ranging from *not at all* to *nearly every day* with higher scores indicating a greater frequency of symptoms.

#### Caregiver well-being

Caregiver well-being was measured using a condensed version of the Psychological Well-Being Scale (C-PWBS; Ryff & Keyes, 1995). The C-PWBS has been used with older adults (Clarke, Marshall, Ryff, & Wheaton, 2001) and caregivers of persons living with dementia (Lethin et al., 2017). The C-PWBS scale is comprised of 24 statements and has moderate reliabilities (α: 0.33–0.56; Ryff & Keyes, 1995). Questions are related to both positive (“I have a sense of direction and purpose in life”; “Most people see me as loving and affectionate”) and negative (e.g., “I often feel overwhelmed by my responsibilities”; “Everyone has their weaknesses, but I seem to have more than my fair share”) aspects of well-being and are answered using a five-point Likert scale ranging from *strongly disagree* to *strongly agree*. Higher scores indicate a greater sense of well-being.

#### Quality of life

The caregiver was asked to rate their quality of life and the quality of life of the care recipient using the Quality of Life in Alzheimer’s Disease scale (QoL-AD). The QoL-AD was originally developed for AD, but has questions relevant to other forms of dementia and has a high reliability (α = 0.86; Logsdon, Gibbons, McCurry, & Teri, 2002). The scale is comprised of 13 items that provide a global assessment of quality of life. The caregiver is asked rate their own quality of life on 13 questions and then to rate the care recipient’s quality of life on the same 13 questions (e.g., “How would you rate [your]/ [your loved one’s] mental health”; “How about [your]/ [your loved one’s] relationship with family members”; “How about [your]/[your loved one’s] ability to do things for fun that [you]/[they] enjoy). Questions are answered using a four-point Likert scale ranging from *poor* to *excellent*. Higher scores indicate a higher perceived level of quality of life.

#### Caregiver social support and network

Caregiver social support was measured using nineteen investigator-generated questions with four dimensions (emotional support, tangible support, affective support, positive social interaction). These questions have been previously validated to use with older adults and have a very high reliability (α = 0.91; Galvin, Scharff, Glasheen, & Fu, 2006). The questions asked participants to rate how often types of social support are available to them (e.g., “Someone you can count on to listen to you when you need to talk”; “Someone to help with daily chores if you were sick”; “Someone to love and make you feel wanted”) using a five-point Likert scale ranging from *none of the time* to *all the time*. Higher scores indicate a greater perception of social support.

The caregiver’s social network was measured using six investigator-generated questions related to the availability and contact frequency that caregivers had with their social network. Question 1 asks participants to rate how many friends they see or hear from at least once a month ranging from *zero* to *nine or greater*. The second question relates to the frequency with which the participant engages with their social support network ranging from *less than monthly* to *daily*. Questions 3 thru 5 are on a on a six-point Likert scale ranging from *never* to *always* and relate to social support (e.g., “When you have an important decision to make do you have someone you can talk to about it”; “Does anybody rely on you for help each day”). Question 6 asks participants if they live alone or with others. Higher scores indicate having and participating in a larger social network.

### Data Analysis

Distributional assumptions were tested to identify outliers; no participants were removed from the analyses due to extreme scores. Six participants were removed because they did not complete the survey beyond the demographic information section. Sample characteristics can be found in Table 1. Analysis of variance was used to examine group differences on continuous variables while chi square statistics were used to examine group differences on categorical variables (SPSS v23; IBM, Armonk, NY). Strength of association between caregiver constructs was examined with Pearson correlation coefficients. Differences between the experience of caregiving as a spouse versus an adult child and by severity of dementia were explored using analysis of covariance (ANCOVA) with caregiver age and primary caregiver status (yes/no) as covariates. There was collinearity between a number of caregiver characteristics (frequency of visits, living with patient, and primary caregiver status); thus, primary caregiver status was selected as the best covariate for ANCOVA analyses. Correction for multiple comparisons was performed using the Bonferroni correction. Post hoc regression analyses were conducted to explore relationship between significant constructs (quality of life, burden, and neuropsychiatric symptoms) seen in adult child caregivers.

## Results

### Sample Characteristics

Sample characteristics are given in Table 1. The majority of caregiver respondents were women (89.1%), white (97.1%), and well-educated (some college and above: 89.4%), but groups did not significantly differ on these characteristics by caregiver status (spouse vs adult child). As expected, adult child caregivers were younger (*p* < .001) and less likely to live with the patient (*p* < .001) or see them on a daily basis (*p* < .001) than spouse caregivers. Adult children caregivers were also more likely to be caring for women care recipients than spouse caregivers who tended to take care of men (34.6% vs 88.6%, *p* < .001). Adult children rated the care recipient’s quality of life lower than spouses (24.9 + 5.2 vs 26.8 + 5.9; *p* < .001), and they cared for older (81.8 + 7.2 vs 71.2 + 8.0, *p* < .001) less educated (*p* < .001), and more impaired (CDR-SB: 11.7 + 4.2 vs 9.7 + 4.1; *p* < .001) care recipients.

In the total sample, caregiver age was strongly correlated with caregiver quality of life (*p* < .001) and well-being (*p* = .002), and inversely correlated with caregiver depression (*p* = .005) and caregiver burden (*p* < .001). Caregiver constructs were highly intercorrelated (Table 2) so that positive attributes (caregiver: well-being quality of life, social support, and social network) were inversely correlated with negative attributes (caregiver: depression, grief, and burden).

**Table 2.** Strength of Association Among Caregiver Self-reported Experience

Variable	Age	Burden	Grief	Depression	Well-being	Quality of life	Social support
Burden	−.195**						
Grief	−.095	.751**					
Depression	−.142**	.519**	.519**				
Well-Being	.159**	−.535**	−.582**	−.573**			
Quality of Life	.286**	−.548**	−.533**	−.619**	.632**		
Social Support	−.087	−.250**	−.393**	−.165**	.386**	.372**	
Social Network	−.040	−.166**	−.231**	−.123*	.365**	.260**	.614**

*Note*: Pearson’s correlation coefficient reported.

*Trend to significance *p* < .05.

**Significant after correction for multiple comparisons *p* ≤ .006.

Relationships between caregiving constructs and ratings of the care recipient’s dementia severity (QDRS, CDR-SB) and frequency of neuropsychiatric symptoms (RMBPC) are given in Table 3. Dementia severity was associated with lower caregiver quality of life and higher caregiver grief and burden. Increasing frequency of neuropsychiatric symptoms was associated with lower caregiver quality of life, and higher depression, grief, and burden. No significant differences between caregiver type (spouse vs adult child) were found on these measures (data not shown).

**Table 3.** Strength of Association Among Caregiving Constructs and Dementia Severity

	QDRS		CDR-SB		NSF	
Variable	*b*	*p*	*b*	*p*	*b*	*p*
Burden	.163	.002**	.104	.04*	.357	<.001**
Grief	.197	<.001**	.160	.002**	.248	<.001**
Depression	.127	.01*	.107	.04*	.161	.002**
Well-Being	−.131	.01*	−.121	.02*	−.092	.07
Quality of Life	−.216	<.001**	−.194	<.001**	−.233	<.001**
Social Support	−.032	.54	−.038	.45	−.102	.05*
Social Networks	−.073	.16	−.073	.15	−.018	.72

*Note*: Regression analysis; Beta values and *p*-values reported; all measures were rated by the caregiver. CDR-SB = Clinical Dementia Rating Sum of Boxes; NSF = Neuropsychiatric symptom frequency as rated on the Revised Memory and Behavioral Problems Checklist; QDRS = Quick Dementia Rating System.

*Trend to significance *p* < .05.

**Significant after correction for multiple comparisons *p* ≤ .007.

### Caregiver Ratings by Caregiver Type

When comparing spouse caregivers to adult child caregivers, a number of notable differences are detected (Table 4). Adult child caregivers report lower quality of life (*p* < .001), and tended to report more caregiver burden, particularly worry about their performance (both *p*’s = .009; not significant when correcting for multiple comparisons). There were no differences in overall caregiver grief, depression, or well-being. Adult child caregivers reported greater social support than spouse caregivers (*p* < .001) across all social support constructs (emotional, tangible, affective, and positive social interaction) without having differences in overall social networks.

**Table 4.** Unadjusted Caregiver Ratings by Caregiver Type

Variable	Spouse	Adult child	*p* value
Burden Total	24.6 (8.3)	26.9 (8.4)	.009*
Role strain	11.7 (3.9)	12.6 (4.5)	.045*
Personal strain	4.4 (2.7)	4.9 (2.7)	.072
Worry about performance	8.6 (3.3)	9.5 (3.2)	.009*
Grief	62.4 (12.9)	60.8 (12.7)	.229
Depression	1.9 (1.6)	2.2 (1.8)	.069
Well-Being	83.5 (12.6)	81.7 (13.0)	.169
Quality of Life	39.0 (7.1)	33.5 (7.6)	<.001**
Social Support Total	57.4 (17.8)	66.8 (20.9)	<.001**
Emotional	25.6 (8.3)	28.1 (9.0)	.006**
Tangible	10.7 (4.7)	13.3 (5.1)	<.001**
Affective	8.9 (3.3)	10.9 (3.6)	<.001**
Social Networks	17.3 (4.5)	18.2 (4.5)	.077

*Note*: Analysis of variance; means, standard deviations and *p* values reported.

*Trend to significance *p* < .05.

**Significant after correction for multiple comparisons *p* ≤ .007.

### Spouse Caregiver Perceptions by Dementia Severity

Within group analyses of spouse caregivers by dementia severity (Table 5) demonstrated that caregivers reported a lower quality of life for the care recipient (*p* < .001) with advancing dementia severity, but not their own quality of life. No other within group differences by severity of disease were seen for spouses.

**Table 5.** Spouse and Adult Child Responses to Caregiving by Disease Severity

	Spouse				Adult Child			
Variable	Mild	Moderate	Severe	*p* value	Mild	Moderate	Severe	*p* value
Patient Age, years	70.3 (8.3)	70.7 (7.9)	72.5 (8.1)	.261	85.3 (96.1)	81.9 (7.5)	81.3 (6.9)	.369
Duration disease, years	2.4 (1.5)	3.3 (1.9)	5.2 (2.8)	<.001**	3.4 (1.7)	2.3 (1.4)	4.7 (2.9)	<.001**
QDRS	7.6 (3.4)	13.1 (4.7)	21.2 (5.3)	<.001**	7.5 (4.7)	14.6 (4.9)	23.1 (4.5)	<.001**
CDR-SB	4.9 (2.5)	8.5 (3.1)	13.9 (3.2)	<.001**	5.2 (3.9)	9.3 (3.3)	14.6 (2.6)	<.001**
Neuropsychiatric Symptom Frequency	27.6 (12.8)	38.0 (13.3)	38.3 (14.5)	.004*	30.3 (5.1)	42.9 (15.9)	41.3 (17.2)	.174
Patient Quality of Life^a^	32.9 (5.8)	27.4 (5.7)	23.4 (4.5)	<.001**	30.1 (4.7)	26.7 (5.6)	22.7 (4.0)	<.001**
Caregiver Age, years	63.0 (7.8)	65.4 (8.9)	67.9 (8.3)	.036*	56.7 (5.3)	52.6 (8.1)	52.3 (7.5)	.349
Caregiver Burden Total	22.6 (10.3)	24.6 (7.7)	25.4 (8.9)	.399	26.5 (8.0)	27.5 (8.6)	26.5 (8.2)	.771
Role Strain	10.2 (4.9)	11.5 (3.6)	12.6 (4.2)	.036*	12.0 (53)	12.7 (4.9)	12.6 (4.5)	.899
Personal Strain	3.9 (2.5)	4.5 (2.6)	4.3 (2.7)	.709	4.8 (1.5)	5.2 (2.6)	4.6 (2.9)	.371
Worry about Performance	8.4 (4.0)	8.6 (3.1)	8.5 (3.5)	.947	9.7 (3.4)	9.5 (3.2)	9.4 (3.3)	.969
Caregiver Grief	59.3 (15.1)	61.9 (12.5)	64.6 (13.2)	.179	57.7 (18.1)	60.1 (12.3)	61.7 (12.7)	.637
Depression	1.7 (1.4)	1.9 (1.6)	2.1 (1.7)	.609	2.9 (2.5)	2.1 (1.8)	2.2 (1.7)	.610
Well-Being	83.5 (11.7)	83.9 (12.5)	82.5 (13.2)	.763	70.0 (22.0)	83.2 (12.8)	81.3 (11.7)	.036*
Caregiver Quality of Life	38.6 (7.9)	37.1 (6.7)	36.4 (7.6)	.461	30.7 (9.3)	34.5 (7.6)	32.9 (7.5)	.264
Social Support Total	53.7 (15.4)	59.9 (17.4)	59.7 (19.4)	.347	54.6 (22.4)	68.3 (21.8)	66.5 (19.8)	.250
Emotional	23.4 (7.9)	25.5 (8.3)	26.7 (8.3)	.272	23.7 (8.2)	28.4 (9.4)	28.2 (8.8)	.424
Tangible	10.1 (4.2)	10.3 (4.6)	11.6 (5.2)	.181	10.7 (6.7)	13.7 (5.2)	13.2 (4.8)	.313
Affective	8.3 (2.7)	8.9 (3.2)	9.1 (3.5)	.659	8.6 (4.9)	11.4 (3.5)	10.7 (3.6)	.121
Positive Social Interaction	11.2 (3.9)	11.1 (4.5)	12.0 (4.5)	.443	11.7 (5.9)	14.7 (4.7)	14.1 (4.8)	.261
Social Network	16.7 (4.3)	17.5 (4.4)	17.2 (4.7)	.710	15.3 (4.2)	18.1 (4.7)	18.5 (4.3)	.187

*Note*: Analysis of covariance; means, SD, and *p*-values reported; disease severity (mild, moderate, severe) characterized by caregiver global ratings of disease severity. CDR-SB = Clinical Dementia Rating Sum of Boxes; QDRS = Quick Dementia Rating System.

^a^Quality of life of the care recipient as rated by the caregiver.

*Trend to significance *p* < .05.

**Significant after correction for multiple comparisons *p* ≤ .003.

### Adult Child Caregiver Perceptions by Dementia Severity

Within group analyses of adult child caregivers by dementia severity (Table 5) demonstrated that caregivers reported a lower quality of life for the care recipient (*p* < .001) with advancing dementia severity, but not their own quality of life. No other within group differences by severity of disease were seen for adult children.

### Between Group Comparisons by Dementia Severity (corrected for multiple comparisons)

Between group analyses of spouse and adult child caregivers by disease severity/stage (Table 6) showed that caregiver grief did not differ in the unadjusted analyses, however when controlling for caregiver age, spouse caregivers experienced greater grief than adult children with advancing dementia severity (*p* = .005).

**Table 6.** Pattern of Significance for Spouse Versus Adult Child Caregiver Responses by Disease Severity

	Unadjusted			Adjusted Model 1			Adjusted Model 2		
Dependent variable	Mild	Moderate	Severe	Mild	Moderate	Severe	Mild	Moderate	Severe
Caregiver Burden Total	.316	.019*	.457	.570	.456	.399	.386	.290	.780
Role Strain	.356	.047*	.889	.519	.666	.107	.266	.235	.520
Personal Strain	.479	.053	.615	.686	.484	.494	.571	.332	.876
Worry about Performance	.424	.059	.099	.605	.557	.757	.730	.686	.826
Caregiver Grief	.787	.340	.184	.492	.018*	.005**	.711	.050*	.041*
Depression	.124	.257	.520	.201	.998	.494	.150	.796	.804
Well-Being	.015*	.691	.575	.045*	.021*	.271	.030*	.450	.574
Caregiver Quality of life	.013*	.011*	.003**	.044*	.859	.683	.014*	.320	.255
Social Support Total	.918	.008*	.035*	.983	<.001**	.006**	.971	<.001**	.047*
Emotional	.939	.017*	.294	.899	.011*	.064	.933	.014*	.259
Tangible	.771	<.001**	.045*	.686	<.001**	.007**	.874	<.001**	.046*
Affective	.873	<.001**	.004**	.001**	<.001**	.002**	.959	<.001**	.018*
Positive Social Interaction	.795	<.001**	.008*	.899	<.001**	.006**	.980	<.001**	.061
Social Network	.483	.371	.081	.484	.225	.044*	.577	.309	.043*

*Note*: Unadjusted analysis of variance and two analyses of covariance (adjusted models); Model 1: adjusted for caregiver age; Model 2: adjusted for caregiver age and primary caregiver status (Y/N); *p* values reported (see Table 5 for means and standard deviations); disease severity (Mild, Moderate, Severe) characterized by caregiver global ratings of disease severity.

*Trend to significance *p* < .05.

**Significant after correction for multiple comparisons *p* ≤ .007.

Adjusted analyses demonstrated that adult child caregivers of care recipients at the moderate to severe stages were better able to increase their social support (tangible, affective, and positive social interaction). In models adjusted for caregiver age (Models 1 and 2) and primary caregiving status (Model 2); these relationships hold true, particularly at the moderate stage of dementia. No differences were seen in other constructs in either unadjusted or adjusted analyses.

### Follow-up Analyses

Regression analyses were conducted post hoc to further explore the relationship between adult child caregivers, neuropsychiatric symptoms and burden. Quality of life for adult children was significantly predicted by caregiver burden (*b* = −.56, *t*(159) = −7.96, *p* < .001); burden also explained a significant proportion of variance in adult child caregivers’ quality of life (*R*^2^ = .30, *F*(1, 159) = 63.36, *p* < .001). Adult child caregiver burden was predicted by the frequency of neuropsychiatric symptoms in the care recipient (*b* = .27, *t*(159) = 3.13, *p* = .002); frequency of neuropsychiatric symptoms in the care recipient also explained a significant proportion of variance in adult child caregiver reported burden (*R*^2^ = .06, *F*(1, 159) = 9.80, *p* = .002).

## Discussion

We hypothesized that spouse and adult child caregivers would experience caregiving for someone with DLB differently, and that score profiles would reveal experiential differences that could guide clinicians to tailor their support for DLB caregivers. Empirically, we found many similarities among the self-reported caregiver experiences of spouses and adult children. In general, perceived levels of burden, grief, depression, and well-being did not significantly differ between spouses and adult children caregivers. Although caregivers each have their own unique experiences, these findings are consistent with a converging opinion that the overall caregiver experience is more similar than different between caregiver type and across diseases.

Despite similarities, there were several differences between spouses and adult children caregivers of DLB. The pattern of DLB caregivers’ quality of life differed significantly between spouses and adult children, with adult children consistently reporting poorer quality of life than spouse caregivers and showing a trend toward reporting more overall burden. Further, we found that burden increased as neuropsychiatric symptoms increased, and that quality of life decreased as burden and neuropsychiatric symptoms increased. In a follow-up analysis, we found that burden predicted adult child caregivers’ quality of life and that adult child caregiver burden was predicted by the frequency of neuropsychiatric symptoms seen in the care recipient. Taken together, this suggests that burden and the frequency of the care recipient’s neuropsychiatric symptoms are likely having a negative impact on adult child caregivers’ quality of life. These findings are consistent with many reports in the literature that neuropsychiatric symptoms in dementia care recipients are among the most stressful aspects of caregiving (Conde-Sala et al., 2010a; Lee, McKeith, Mosimann, Ghosh‐Nodyal, & Thomas, 2013; Leggett, Zarit, Taylor, & Galvin, 2011; Papastavrou, Kalokerinou, Papacostas, Tsangari, & Sourtzi, 2007; Rinaldi et al., 2005; Schulz & Sherwood, 2008) and that the amount of neuropsychiatric symptoms exhibited by the care recipient is associated with the amount of self-reported dementia caregiver burden (Holley & Mast, 2010; Leggett et al., 2011; Papastavrou et al., 2007; Schulz & Sherwood, 2008).

DLB tends to be a male-predominant disease; interestingly, adult child caregivers in this sample were more likely to be daughters caring for their mothers, and spouse caregivers were more likely to be wives caring for their husbands. Although adult child caregivers were younger on average, they were more likely to take care of older, more severely affected persons with DLB that were in the later stages of the disease when compared with spouses. Adult children were also not in contact with the care recipient as often as spouses, and were more likely to have the patient in a skilled facility. These findings are consistent with reports that adult children are less involved in daily care (Ankri, Andrieu, Beaufils, Grand, & Henrard, 2005) and more likely to place the care recipient in a skilled facility (Montgomery & Kosloski, 1994). Even though adult children had less exposure to their care recipient, the intermittent exposure to the neuropsychiatric features of DLB, resulted in self-reports of poorer quality of life. Because this was a retrospective survey delivered online, we have no way of knowing the objective amount of caregiving delivered in real time, so we cannot know if this effect is a function of limited exposure to neuropsychiatric features or some other type of caregiver reaction (either minimizing in spouse self-reports or catastrophizing in adult children).

The older our participants, the higher their well-being and quality of life. This is consistent with previously reported findings that older people, when compared with younger people, report fewer negative emotional experiences and being more satisfied with their life (Anderson et al., 2013; Gross et al., 1997). In our model, quality of life was significant until age was statistically controlled. Thus, age may be acting as a buffer against deleterious aspects of caregiving.

Spouses and adult children in our sample reported similar levels of grief that grew more severe as the care recipient’s DLB progressed; however, spouses reported greater grief at the most severe disease stage when compared to adult children in the age adjusted model. These findings align well with a study of AD caregivers that found the amount of caregiver grief increased as the care recipient’s disease course progressed, and that no difference was found between spouses and adult children when the care recipient lived in the family home, but, once the care recipient was placed in a facility outside the home (typically when the disease is more progressed), spouses reported more grief than adult children (Ott et al., 2007).We did not find a significant difference between spouses and adult children in depression and well-being, this pattern of scores diverges from the AD caregiver literature that usually finds spouses experience more depression (Covinsky et al., 2003; Lou et al., 2015; Pinquart & Sörensen, 2011; Schulz & Sherwood, 2008) and less well-being (Anderson et al., 2013; Conde-Sala et al., 2010b) than do adult children. This may be due to differences in measurement scales for these constructs, or represent differences in the caregiving experience for DLB. More research on caregiving for DLB is needed to further elucidate these finding.

Adult child caregivers reported more social support overall and across disease stages. They were also more effective than spouse caregivers at increasing their social support (across multiple domains: emotional, tangible, and affective) as the care recipient’s disease progressed, without significantly increasing the size of their social network. It may be that adult children enriched their network without recruiting new friends, presumably by increasing their intimacy and reliance on their existing network. Or, it may be that the content of their social network changed (e.g., relying more on other types of social support to help with caregiving: social workers, doctors, nurses vs family and friends), but the size remained the same.

Although higher social support and social engagement have been associated with higher levels of life satisfaction (Haley et al., 2003; Hämmerling et al., 2008), this was not case in this sample of DLB caregivers. While adult children endorsed using more social support than spouse caregivers, spouse caregivers are paradoxically reporting a higher quality of life than adult children. A qualitative study found that spouse caregivers tended to view the sacrifice of their leisure and social activities as a necessary provision of care, while adult children tended express frustration and bitterness (Loos & Bowd, 1997). Spouses may have more time to dedicate to caregiving than adult children. Adult children are more likely than spouse caregivers to be juggling multiple roles (e.g., DLB caregiver, spouse, parent, career), and the burden of their care responsibilities makes it difficult for them to reconcile roles (Beitman et al. 2004; Stephens, Townsend, Martire, & Druley, 2001; Wang, Shyu, Chen, & Yang, 2011). While adult children were shown to increase their social support as the care recipient’s disease stage increased, their ability to engage in leisure activities and to fulfill their responsibilities outside of caregiving was most likely undermined as they spent more time on caregiving duties. It may be that adult children are experiencing negative reactions to the changes in their life that have occurred as a result of assuming the caregiving role, and that these feelings are contributing to a lower quality of life for adult children caregivers of DLB.

### Future Directions and Limitations

This study is focused on the self-reported caregiving experience of spouse and adult child caregivers of individuals with DLB; thus, direct comparisons with other dementia types was not possible. DLB caregivers have been found to report more burden and a lower quality of life than AD caregivers (Svendsboe et al., 2016; Thomas et al., 2006). Future research can compare caregivers of DLB with other dementia populations. This was a cross-sectional study of DLB. Longitudinal studies of DLB caregivers would provide more information about how the caregiving experience changes overtime. One limitation of this study is that female caregivers were over-represented in this sample. Recruiting men has been a particular challenge for the caregiving literature, as caregivers are more typically female (Ivery & Muniz, 2017). DLB tends to be a male-predominant condition, which makes recruiting male caregivers even more of a challenge. Still, future studies of DLB caregivers should attempt to recruit more male caregivers, because it has been found that being a male caregiver was associated with a greater sense of well-being and the reporting of a lower perception of burden when caring for those with dementia (Papastavrou et al., 2007). Another limitation of this study is that those who chose to participate were predominately white; being African American has been shown to be associated with greater sense of well-being among dementia caregivers. Finally, the number of hours of care was not collected as a part of this study; the number of hours of care has been shown to be a predictor of caregiver burden (Kim, Chang, Rose, & Kim, 2012; Pinquart & Sörensen, 2011).

## Implications

Spouses and adult children experience caregiving for those with DLB differently; this can be explained in part by the younger age of the adult child caregiver, the frequency with which they see the care recipient, and the more severe stage of the care recipient. Overall, we found that spouse and adult child caregivers of DLB report similar levels of burden, grief, depression, and well-being. However, there are important differences overall and by stage of dementia between caregiver groups in caregiver quality of life, social support, and caregiver grief. Spouse caregivers report a greater quality of life and experience more grief at the most severe disease stage.

Despite social support typically providing a buffer against caregiver burden and depression, resulting in higher quality of life and a greater sense of well-being, adult child caregivers in this study perceived receiving more social support and reported a lower quality of life than spouse caregivers of DLB. Adult children were also showing a trend toward reporting more burden than spouses, and, in follow-up analyses, we found that adult child caregivers’ quality of life was significantly predicted by burden, and that burden was significantly predicted by the frequency of neuropsychiatric symptoms in the care recipient. These findings suggest that burden and the frequency of the care recipient’s neuropsychiatric symptoms are negatively impacting adult child caregivers’ quality of life.

DLB caregiver support for this population should target psychoeducation for complicated neuropsychiatric symptoms seen in the care recipient, as the experience of increasing caregiver burden, grief, and depression were associated with growing neuropsychiatric symptom profiles in the care recipient. A meta-analysis of nonpharmacological interventions for dementia found that psychoeducation that focuses on improving caregiver’s understanding of the illness, self‐care, and patient care, may be as effective, if not more effective than medication at reducing caregivers’ distress and care recipients’ neuropsychiatric symptoms (Brodaty & Arasaratnam, 2012). Thus, it is important to treat the care recipient’s neuropsychiatric symptoms as a way to benefit both the care recipient and their caregiver. Clinically significant depression among dementia caregivers is also common (Watson et al., 2011). A recent study comparing AD and DLB caregivers found that, DLB caregivers reported more burden than AD caregivers, and 31% of DLB caregivers were at risk of clinically relevant distress (indicating that they would require additional referral and support; Svendsboe et al., 2016). Therefore, screening all DLB caregivers for burden, grief, and depression is suggested to identify those that may benefit most from intervention.

An area of possible intervention for spouses specifically is in increasing social support (Dam, de Vugt, Klinkenberg, Verhey, & van Boxtel, 2016), and providing more referrals to community-based services (Dang, Badiye, & Kelkar, 2008). A recent systematic review of social support interventions for dementia caregivers found that social support interventions improve caregiver well-being and depression (Dam et al., 2016), and may be especially beneficial to spouse caregivers of DLB. Adult children may benefit most from interventions aimed at mitigating burden and improving quality of life.
